# Magnetic Concentric Hot-Circle Generation at Optical Frequencies in All-Dielectric Mesoscale Janus Particles

**DOI:** 10.3390/nano12193428

**Published:** 2022-09-30

**Authors:** Oleg V. Minin, Song Zhou, Cheng-Yang Liu, Jelene Antonicole Ngan Kong, Igor V. Minin

**Affiliations:** 1 Nondestructive Testing School, Tomsk Polytechnic University, 30 Lenin Ave., Tomsk 634050, Russia; 2 Jiangsu Key Laboratory of Advanced Manufacturing Technology, Faculty of Mechanical and Material Engineering, Huaiyin Institute of Technology, Huai’an 223003, China; 3 Department of Biomedical Engineering, National Yang Ming Chiao Tung University, Taipei City 11221, Taiwan; 4 Medical Device Innovation and Translation Center, National Yang Ming Chiao Tung University, Taipei City 11221, Taiwan

**Keywords:** Janus particle, hot-spot generation, magnetic field, magnetic hot circles, mesotronics

## Abstract

The development of all-dielectric structures with high magnetic response at optical frequencies has become a matter of intense study in past years. However, magnetic effects are weak at optical frequencies due to the small value of the magnetic permeability of natural materials. To this end, natural dielectric materials are unemployable for practical “magnetic” applications in optics. We have shown for the first time that it is possible to induce intense magnetic concentric subwavelength “hot circles” in a dielectric mesoscale Janus particle. The basis of the Janus particle is a combination of the effects of a photonic jet, whispering-gallery waves, and the concept of solid immersion. Simulations show an (H/H_0_)^2^/(E/E_0_)^2^ contrast of more than 10, and maximal magnetic field intensity enhancement is more than 1000 for a wavelength-scaled particle with a refractive index *n* < 2 and a size parameter in the order of 30. This work may provide a new way to realize precise magnetic devices for integrated photonic circuits and light–matter interaction.

## 1. Introduction

The strong localization of optical waves to volumes below the diffraction limit is a topic of extensive research involving a wide range of applications [1,2,3,4,5]. The ability to localize an optical wave to sub-wavelength volumes is called hot-spot engineering [6]. Through manipulation, the intensities of both the electric and magnetic fields can be enhanced to up to several orders of magnitude. A single dielectric spherical nanoparticle with high permittivity can exhibit a strong electromagnetic resonance [7], of which the first fundamental mode corresponds to magnetic dipole excitation, but the fabrication tolerances must be tailored down to the sub-nanometer resolution. The generation of strong magnetic hot spots by dielectric nanoparticles was first observed in inter-particle regions [8,9,10]. The interference of the magnetic and electric modes in such nanoparticle assemblies gives rise to sharp magnetic Fano resonances [11,12,13,14]. Dielectric wavelength-scaled (mesoscale) particles with a Mie-sized parameter *q = kR*, where k is the wavenumber and R represents particle radius, to the order of *q ~* 10 have aroused big interest because of their potential to localize light at the sub-wavelength scale [15,16] and because of their ability to yield high internal magnetic and electric local field enhancements instead of plasmonic metal nanoparticles [17,18,19]. Moreso, the employment of mesoscale dielectric particles has facilitated the achievement of the remarkable magnetic enhancement of overcoming the inherent losses of plasmonic materials. Optical magnetic field localization squeezes into deep sub-wavelength regions, which opens promising perspectives for spintronics [20].

Recently, it has been shown that the Mie-type resonances of different orders overlap by increasing the refractive index to be greater than 1.4 [21]. In effect, this leads to a higher concentration of the electric and magnetic fields being focused within low-loss dielectric spherical particles with diameters less than the incident wavelength. For spherical gallium phosphide particles with a refractive index of *n* = 3.4932 at the specified wavelength of 532 nm and a Mie-sized parameter of *q* = 5.38, maximal field intensities of E^2^ ~ 40 and H^2^ ~ 140 were observed. In another study, a high-resonance effect was reported when using a particle with *n* = 1.46 and *q* ~ 37 [22]. It was observed that one of the resonant scattering coefficients was 20 times higher in magnitude than the other coefficients. This abnormal value was described as the constructive interference of one partial wave inside the microsphere. Later, the optical “super-resonance effect” in mesoscale dielectric spheres based on the high-order Fano resonance and caused by the particle’s internal partial waves was proposed [23,24,25]. In theory, this effect allows the attainment of super-high intensity magnetic fields. It is valid for a range of Mie-sized parameters *q ~* 10–70 and a refractive index of *n* < 2, which theoretically render a field-enhancement effect that is more than 10^7^ times stronger than that of downward radiation [26]. Moreover, it demonstrated the possibility of overcoming the diffraction limit despite having high sensitivity to losses in the particle material. Additionally, an unusual effect—the hot spot size decreasing down to less than the immersed diffraction limit of the particle material, with a tiny change being observed after the introduction of small dissipation into the particle material—was observed for the first time [24].

While the shapes of the mesoscale dielectric spherical particles have only 2 degrees of freedom (Mie-sized parameter *q* and refractive index *n* of the particle material), optically asymmetric particles or particles with broken spherical or cylindrical symmetries, called Janus particles [27], provide additional degrees of flexibility in electromagnetic response tuning [28,29]. While shaping the high-order Fano resonance has created opportunities for localized magnetic and electric field manipulation, we have proposed a more fundamental approach [29]. By tailoring the broken symmetry of the spherical- or cylindrical-shaped particles, we can facilitate new kinds of localization and enhancement of the electromagnetic fields’ hot spots inside Janus particles near their shadow surface. The introduction of broken symmetry into dielectric spherical or cylindrical particles as an additional degree of freedom enlarges the capacity for strong field localization beyond the diffraction limit at the nanoscale, opening a room of opportunities for new applications. In this manner, we find that spherical or cylindrical dielectric mesoscale particles with broken symmetry can generate stable nanoscale hot-spots with giant field intensity enhancement.

## 2. Computational Model

**Figure 1:**
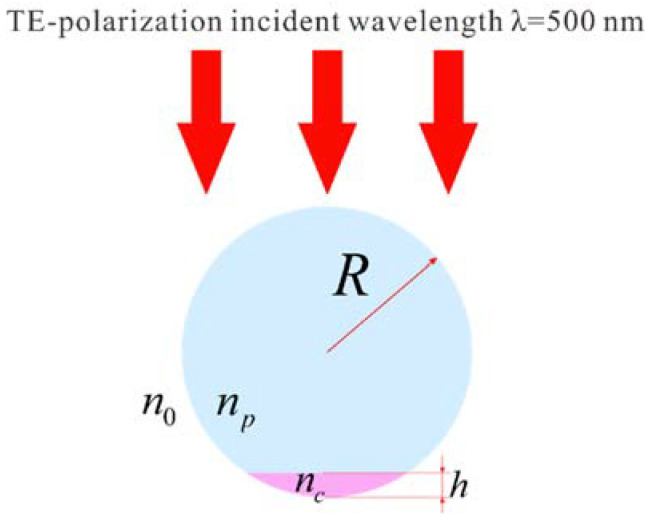
A mesoscale Janus particle illuminated under a TE-polarization plane wave.

Magnetic concentric hot-circle generation at optical frequencies in all-dielectric mesoscale Janus particles is investigated using the wave optics module of COMSOL Multiphysics, a commercial finite element software. As seen in the schematic of the model shown in Figure 1, the Janus particle is formed by a cut cylinder with a truncation height *h*, where the radius of the cylinder is defined as R. The Janus particle is illuminated under a TE-polarization plane wave with the incident wavelength λ = 500 nm. The refractive index of the upper part of the Janus particle is set to *n_p_* = 1.5, while that of the bottom part is chosen as *n_c_*. The particle is surrounded by medium with a refractive index of *n_0_* = 1. In the simulation, an incident TE-polarized plane wave with E_0_ = 1 is added into the wave optics module as a background electric field, and perfectly matched layer-absorbing boundary conditions are utilized around the computational domain. To guarantee the accuracy of the simulation, the maximum element size of the free triangular mesh is set to 5 nm in the bottom part of the Janus particle and to 20 nm in the other computational domains. The electric intensity enhancement is defined as (E/E_0_)^2^, and the magnetic intensity enhancement is defined as (H/H_0_)^2^, where H_0_ = √ε_0_/μ_0_ × E_0_.

## 3. Simulation and Results

**Figure 2:**
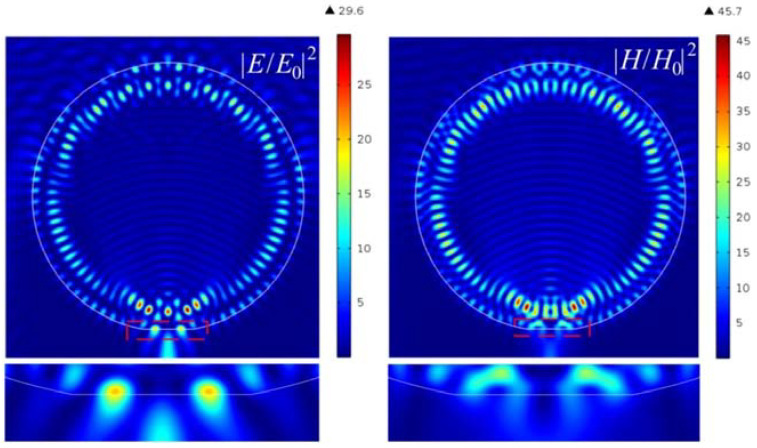
Hot-spot generation in cylindrical Janus particle with parameters *h* = 42 nm and *n* = 1.5 (refractive index of the cutting area: *n_c_* = 1).

The main idea of the new field localization mechanism in the Janus particle is the combination of the effects of photonic nanojet and whispering-gallery waves. At a fixed truncation height *h*, sharp resonances are observed in the intensities of the electric and magnetic fields as a function of the Mie-sized parameter *q*. Approximately the same resonance distributions are observed in the case of high Fano resonances [23]. With a change in the truncation height *h* and when the vector H of the incident plane wave lies in the *x-y* plane of the truncated element of the sphere, a narrow resonance is observed for the TM mode. In this case, hot-spots with an extremely high intensity appear on the flat surface of the truncated element. These are associated with the excitation of the whispering-gallery waves on the flat element of the truncated surface [29]. Considering a cylindrical particle with the following main parameters: cylinder radius *R* = 5λ, which corresponds to the resonant size parameter of *q* = 31.41593 at a wavelength of λ = 500 nm, and a particle material refractive index of *n_p_* = 1.5. These are the usual particle parameters for the formation of a photonic jet. Below, the refractive index contrast (*n* = *n_p_*/*n_c_*) is the ratio of the particle material *n_p_* to the cutting-area material *n_c_*. Figure 2 clearly shows the “electric” photonic jet and two hot spots near the flat boundary of the Janus particle. Detailed studies of the resonance properties of such a Janus particle are given in previous literature [29].

**Figure 3:**
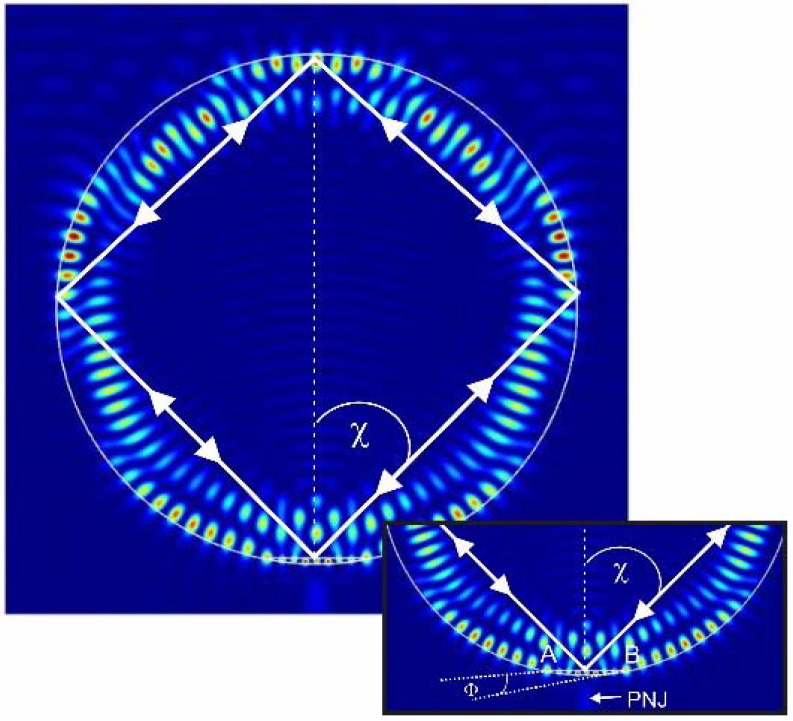
The path of the rays in a Janus particle in the case of a ray falling on a flat surface at an angle of total internal reflection *χ*. The inset shows a schematic change in the phase of a wave along a section of a flat surface.

The work of the Janus particle can be clearly explained based on the geometric–optical approximation shown in Figure 3 [30]. When radiation is incident at an angle of total internal reflection $\chi_{0}=\arcsin(n_{m}/n_{p})$, flat surfaces play the role of a mirror. The interference of the waves incident on a flat surface at angles of total internal reflection creates high-intensity evanescent fields near the surface, for example, at *n* = 1.5 and $\chi_{0}≅41.8^{0}<45^{0}$; however, for small truncations of a cylindrical particle, the first resonance should be observed at *χ* ≈ π/4 = 45°. The difference in the angle value χ is explained by the fact that on the line of the intersection of a flat section with a cylindrical surface, a wave phase-jump occurs, which can be determined from the generalized law of refraction [30,31,32]:

(1) $$n_{p}\sin\theta_{p}-n_{m}\sin\theta_{m}=\frac{\lambda }{2\pi }\frac{d\Phi }{dx}$$

where dΦ/dx is the change in the phase gradient of the wave depending on the height of the truncated element *h*.

In this case, the angle of total internal reflection changes as [30]:

(2) $$\chi =\arcsin(\frac{n_{m}}{n_{p}}+\frac{\lambda }{2\pi n_{p}}\frac{d\Phi }{dx})$$

Note that for small truncations *h*, the correction Δ*χ* to the angle of total internal reflection $\chi_{0}$ is proportional to the height of the truncated element *h* and is inversely proportional to the refractive index *n_p_*: *χ* ≈ *χ*_0_ + Δ*χ*, Δ*χ* = *βh*/*n_p_q*, and *β* = constant. The development of the Janus particle consists of the involvement of the concept of solid immersion integrated onto a dielectric particle. The particle consists of two parts, the main lower and the smaller upper parts, both of which have different refractive indices. The high-index material in the smaller upper part allows new Janus particles to access contributions from the solid immersion mechanism [33].

**Figure 4:**
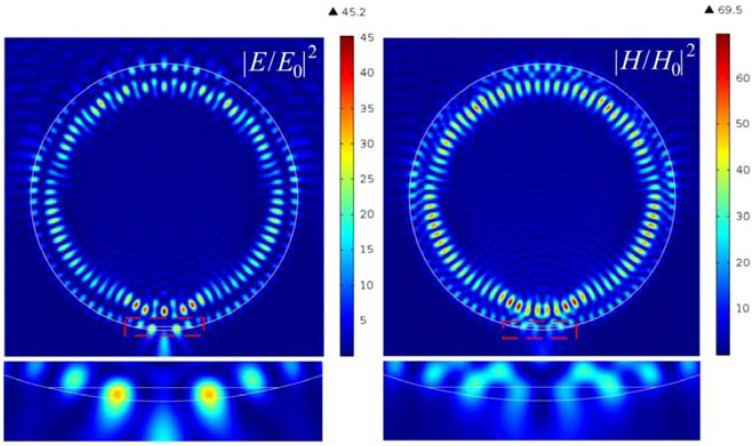
Hot-spot generation in cylindrical Janus particle with the parameters *h* = 68 nm and *n* = 1.1236 (water).

**Figure 5:**
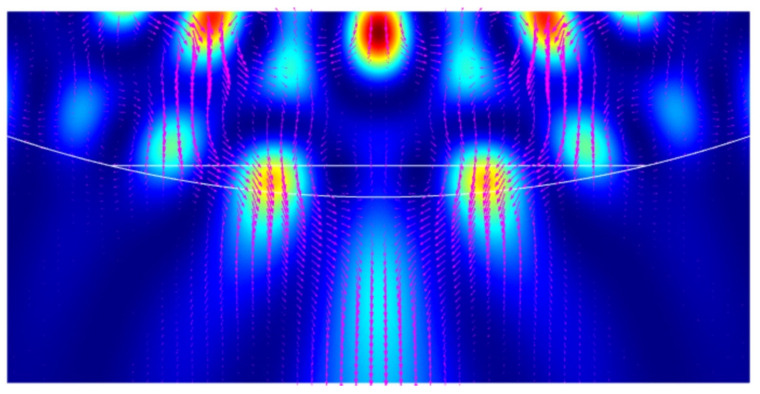
Distribution of the Poynting vector around the hot spots of the Janus particle.

Figure 4 shows the generation of hot spots when the truncated portion of the cylinder is filled with water. Since the refractive index of water is intermediate between the refractive index of the particle and vacuum, this part of the particle acts as a dielectric matching layer that reduces reflection from a flat surface [34]. The formation of a photonic jet, in this case, is due to the specific distribution of the hot spots and vortices [26,30] inside the particle and the low-index dielectric layer in its shadow portion, which is shown in Figure 5.

**Figure 6:**
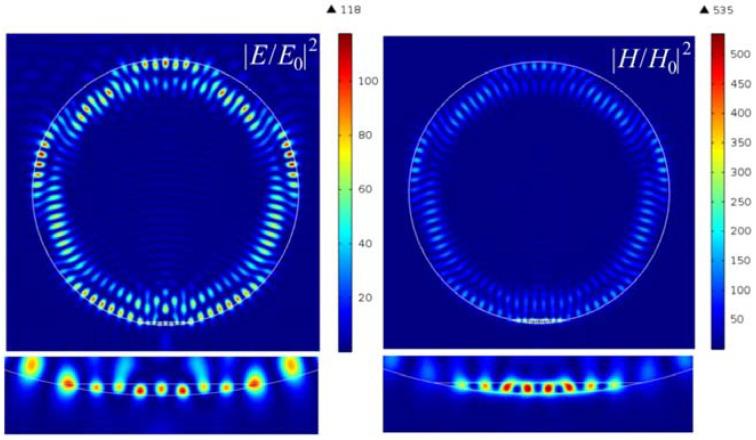
Hot-spot generation in cylindrical Janus particle with the parameters *h* = 58 nm and *n* = 0.476.

With an increase in the contrast of the refractive index and in the dielectric layer with a high refractive index, zones of hot spots with high intensities are formed, as shown in Figure 6. By comparing the field intensity distribution in Figure 2 and Figure 6, the two-material composite cylindrical Janus particle has more converged hot spots than the initial configuration in Figure 2. Moreover, one can see that the multiple localized hot spots are in an annular arrangement across the cylindrical boundary, which is due to the cylindrical symmetry of the Janus particle. Additionally, several higher enhancements appear inside of the internal high-index material, which are caused by wave interference at two material interfaces.

**Figure 7:**
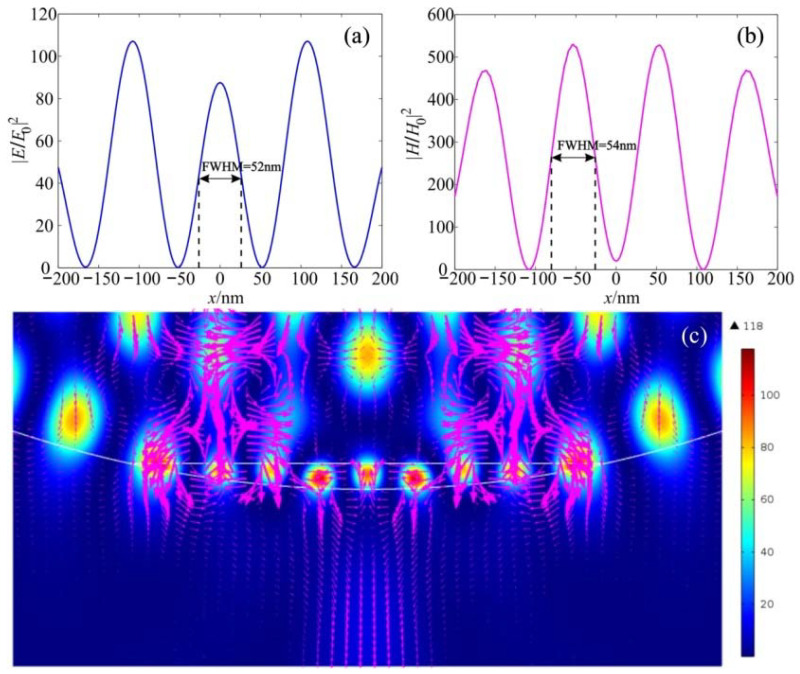
Field intensity distribution along the extreme hot spots for the (**a**) electric and (**b**) magnetic components; (**c**) Poynting vector energy flux.

Figure 7 shows the field intensity distribution along the extreme hot spots for the electric and magnetic components. Figure 7c shows the vortices and the Poynting vector’s energy flux near the hot spots, demonstrating complex vortex flow in this area. It can be observed that the half-width of the intensity maximum for both the electric and magnetic fields is about 0.11λ, which is much smaller than the solid immersion limit criterion. In this case, the enhancement of the intensity of the magnetic field is about 500, which is about 4–5 times higher than that of the electric field.

**Figure 8:**
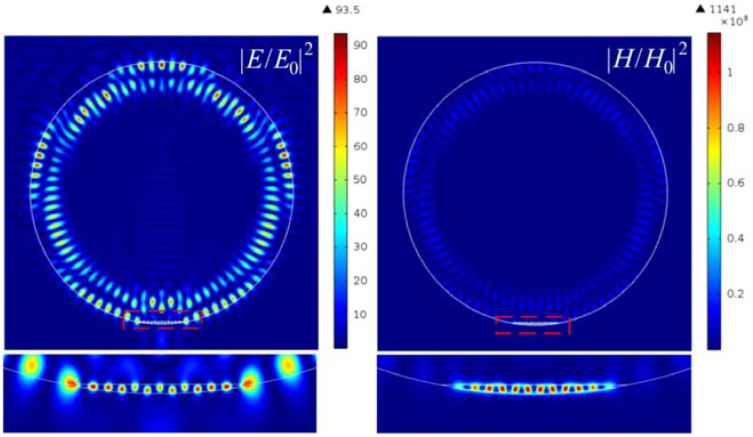
Hot-spot generation in cylindrical Janus particle with the parameters *h* = 46 nm and *n* = 0.3.

**Figure 9:**
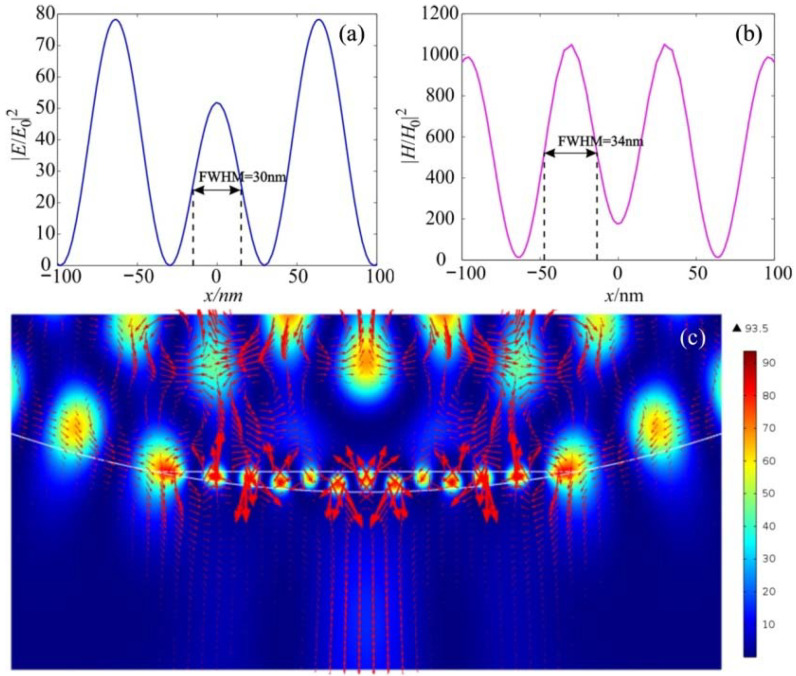
Field intensity distribution along the extrema of the hot spots for the (**a**) electric and (**b**) magnetic components; (**c**) Poynting vector energy flux.

A further increase in the refractive index of the material of the truncated cylinder led to an even greater increase in the intensity of the hot spots of the magnetic field, as shown in Figure 8 and Figure 9. It can also be seen that the half-width of the intensity maximum for both the electric and magnetic fields is about 0.064λ, which is also much smaller than the solid immersion limit criterion. In this case, the enhancement of the intensity of the magnetic field is about 1000, which is about 12 times greater than that of the electric field.

**Figure 10:**
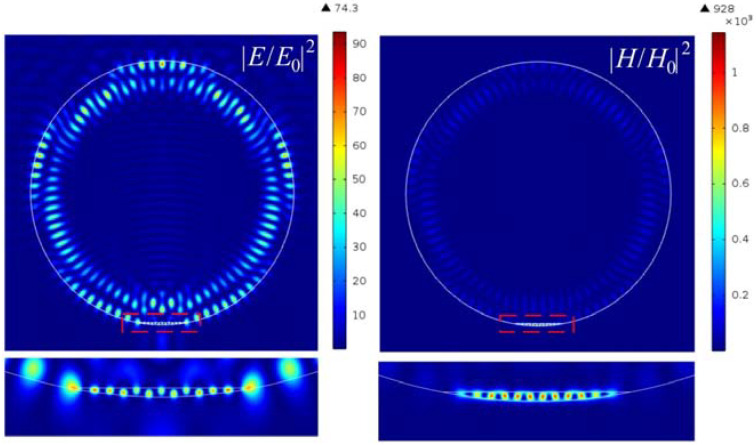
Hot-spot generation in cylindrical Janus particle with the parameters *h* = 46 nm, *n* = 0.3, and *k* = 0.005.

**Figure 11:**
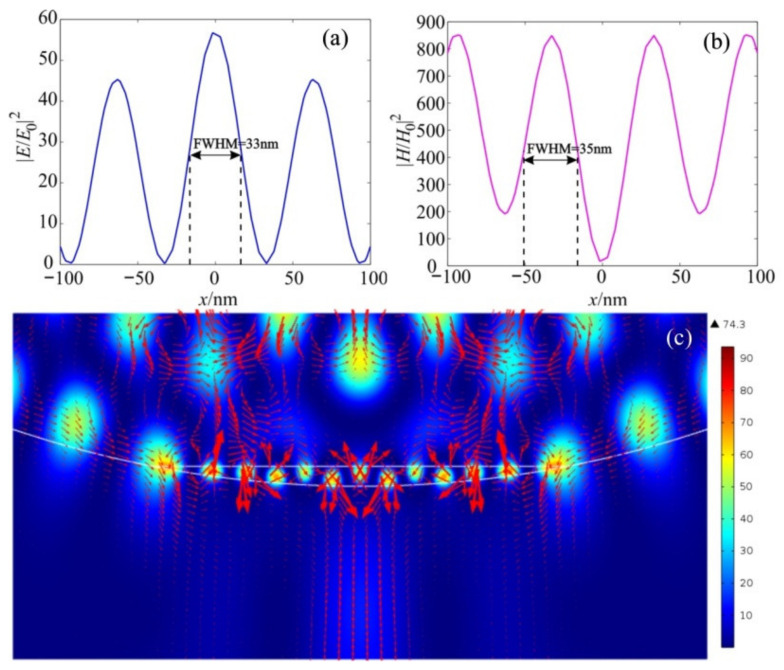
Field intensity distribution along the extrema of the hot spots for (**a**) electric and (**b**) magnetic components; (**c**) Poynting vector energy flux.

It is known that “super resonances” are quite sensitive to dissipation [23,24,25,26,30]. With low dissipation, these resonances are strongly suppressed. In Figure 10 and Figure 11 below, the generation of magnetic hot spots for particles with reference index contrast of *n* = 0.3 are shown. In these figures, however, the material used for the dielectric on the bottom side is called Rhenium Diselenide (ReSe_2_), which has a reference index near 5 and losses of *k* = 0.005 [35,36,37]. Note that the values of the magnetic field intensity for *n* = 0.3 are approximately two times higher than those of the spherical particles without losses [21]. Comparative characteristics of the hot spots of Janus particles are presented in Table 1. The introduction of losses into the dielectric material led to a drop in the intensity of the electric field by almost 20% and of the magnetic field by 18%.

## 4. Conclusions

The generation of deep subwavelength magnetic hot spots in mesotronics [38] based on a new physical principle, aside from their key role in fundamental physics, provides a new degree of freedom for all-dielectric mesoscale structures, which can control unconventional photonic processes. Consequently, artificial optical magnetism is an active topic of research, and great attention has been devoted to all dielectric wavelength-scaled structures that generate magnetic hot spots. We have shown that it is possible to induce intense magnetic concentric subwavelength hot circles in a dielectric mesoscale Janus particle. The basis of the Janus particle is a combination of the effects of a photonic jet, whispering-gallery waves, and the concept of solid immersion. Applying morphological symmetry breaking on the cylindrical particle, we could switch from electric-field hot spots to magnetic hot spots with field enhancement of up to multiple orders of magnitude. As expected, magnetic and electrical hot spots are sensitive to losses in the dielectric material. Simulations show an (H/H_0_)^2^/(E/E_0_)^2^ contrast of more than 10 for a wavelength-scaled particle with refractive index *n* < 2 with an optimized depth of the high-index layer that escalates as the Mie-sized parameter increases. For such Janus particles, conventional nonlinear optics related to nonlinearity ε = ε(E) is dominant. The proposed generation method for magnetic hot spots is prospectively useful for magneto–optical devices in photonic applications, for enhancing magnetic light–matter interaction from quantum computing [39] to sensing [40], maser [41], nanoparticle trapping [42], and in superlensing, spintronics, nonlinear spectroscopy, magnetic recording [43,44], etc.

## Figures and Tables

**Table 1:** Comparative characteristics of hot spots of Janus particles.

*n*	(H/H_0_)^2^	(E/E_0_)^2^	(H/H_0_)^2^/(E/E_0_)^2^
1.5	45	29	1.55
1.124	69	45	1.53
0.476	535	118	4.53
0.3 (*k* = 0)	1141	93	12.27
0.3 (*k* = 0.005)	928	74	12.54

## Data Availability

The data that support the findings of this study are available from the corresponding author upon reasonable request.
